# Nurses' Remuneration and Pensions

**Published:** 1886-11-20

**Authors:** 


### The Editor's Letter-Box.

[Our Correspondents are reminded that prolixity is a great bar to publication, and that brevity of style and conciseness of statement greatly facilitate early publication.]

### NURSES' REMUNERATION AND PENSIONS: A VOICE FROM AMERICA.

Miss Alice Fisher, superintendent of nurses at the Philadelphia Hospital, West Philadelphia, writes: "I understand that a plan has been set on foot in England, which has been greatly promoted by you, for the purpose of instituting a General Nurses' Annuity and Sick Helping Society. If this is the case, how can I obtain any papers in connection with it, or other information? I am very desirous that something of the same kind should be started here, but I should like to know all I can about other people's plans first. One of the clergy of the Episcopal Church came to me the other day with the beginning of a scheme, but it was intended only for the benefit of Church women, which, though a Church woman myself, I could not agree to. I believe a well-organised society would be joined by an immense number of nurses here. I am in charge of a large city hospital of twelve hundred beds, and have had very hard work for two years, but I am thankful to say I am more than repaid by the result of my labours. No one accustomed only to English hospital life could understand the difficulties we have to overcome on this side. Nevertheless, though we have every possible disadvantage of building, etc., to contend with, our patients prosper and our nurses are sought after all over America. I have fifty-two nurses always in the training school, half of them being unpaid, which is a novelty in America, but which seems to succeed, and, indeed, attracts a superior class of women to those I see in other hospitals. The wages given to private nurses are very high. I can, for example, always get for a good surgical or nervous nurse $25; that is to say, £5 a week. I believe it would answer for a great many English women to come out here to nurse, if they had a little money and could wait a certain time. The American women most suitable for nurses become doctors; but I believe very few of them are able to support themselves, and I think, also, the demand for women doctors is becoming less, so that I should not be surprised by-and-by to recruit many for my own profession from their ranks."

